# Three-dimensional intracardiac echocardiography for left atrial appendage sizing and percutaneous occlusion guidance

**DOI:** 10.1093/europace/euae010

**Published:** 2024-01-16

**Authors:** Domenico G Della Rocca, Michele Magnocavallo, Carola Gianni, Sanghamitra Mohanty, Amin Al-Ahmad, Mohamed Bassiouny, Marialessia Denora, Vincenzo Mirco La Fazia, Carlo Lavalle, Gerald J Gallinghouse, Pasquale Santangeli, Marco Polselli, Andrea Sarkozy, Giampaolo Vetta, Adnan Ahmed, Javier E Sanchez, Luigi Pannone, Gian-Battista Chierchia, David R Tschopp, Carlo de Asmundis, Luigi Di Biase, Dhanunjaya Lakkireddy, David J Burkhardt, Rodney P Horton, Andrea Natale

**Affiliations:** Texas Cardiac Arrhythmia Institute, St. David’s Medical Center, 3000 N Interstate Hwy 35 Suite 720, Austin, TX, USA; Heart Rhythm Management Centre, Postgraduate Program in Cardiac Electrophysiology and Pacing, Universitair Ziekenhuis Brussel-Vrije Universiteit Brussel, European Reference Networks Guard-Heart, Av. du Laerbeek 101, 1090 Jette, Brussels, Belgium; Texas Cardiac Arrhythmia Institute, St. David’s Medical Center, 3000 N Interstate Hwy 35 Suite 720, Austin, TX, USA; Arrhythmology Unit, Ospedale San Giovanni Calibita, Fatebefratelli Isola Tiberina—Gemelli Isola, Rome, Italy; Texas Cardiac Arrhythmia Institute, St. David’s Medical Center, 3000 N Interstate Hwy 35 Suite 720, Austin, TX, USA; Texas Cardiac Arrhythmia Institute, St. David’s Medical Center, 3000 N Interstate Hwy 35 Suite 720, Austin, TX, USA; Texas Cardiac Arrhythmia Institute, St. David’s Medical Center, 3000 N Interstate Hwy 35 Suite 720, Austin, TX, USA; Texas Cardiac Arrhythmia Institute, St. David’s Medical Center, 3000 N Interstate Hwy 35 Suite 720, Austin, TX, USA; Texas Cardiac Arrhythmia Institute, St. David’s Medical Center, 3000 N Interstate Hwy 35 Suite 720, Austin, TX, USA; Texas Cardiac Arrhythmia Institute, St. David’s Medical Center, 3000 N Interstate Hwy 35 Suite 720, Austin, TX, USA; Department of Clinical, Internal, Anesthesiologist and Cardiovascular Sciences, Sapienza University of Rome, Viale del Policlinico 155, 00161 Rome, Italy; Texas Cardiac Arrhythmia Institute, St. David’s Medical Center, 3000 N Interstate Hwy 35 Suite 720, Austin, TX, USA; Section of Cardiac Pacing and Electrophysiology, Department of Cardiovascular Medicine, Cleveland Clinic, Cleveland, OH, USA; Arrhythmology Unit, Ospedale San Giovanni Calibita, Fatebefratelli Isola Tiberina—Gemelli Isola, Rome, Italy; Heart Rhythm Management Centre, Postgraduate Program in Cardiac Electrophysiology and Pacing, Universitair Ziekenhuis Brussel-Vrije Universiteit Brussel, European Reference Networks Guard-Heart, Av. du Laerbeek 101, 1090 Jette, Brussels, Belgium; Heart Rhythm Management Centre, Postgraduate Program in Cardiac Electrophysiology and Pacing, Universitair Ziekenhuis Brussel-Vrije Universiteit Brussel, European Reference Networks Guard-Heart, Av. du Laerbeek 101, 1090 Jette, Brussels, Belgium; Kansas City Heart Rhythm Institute, 5100 W 110th St Second Floor, Overland Park, KS, USA; Texas Cardiac Arrhythmia Institute, St. David’s Medical Center, 3000 N Interstate Hwy 35 Suite 720, Austin, TX, USA; Heart Rhythm Management Centre, Postgraduate Program in Cardiac Electrophysiology and Pacing, Universitair Ziekenhuis Brussel-Vrije Universiteit Brussel, European Reference Networks Guard-Heart, Av. du Laerbeek 101, 1090 Jette, Brussels, Belgium; Heart Rhythm Management Centre, Postgraduate Program in Cardiac Electrophysiology and Pacing, Universitair Ziekenhuis Brussel-Vrije Universiteit Brussel, European Reference Networks Guard-Heart, Av. du Laerbeek 101, 1090 Jette, Brussels, Belgium; Texas Cardiac Arrhythmia Institute, St. David’s Medical Center, 3000 N Interstate Hwy 35 Suite 720, Austin, TX, USA; Heart Rhythm Management Centre, Postgraduate Program in Cardiac Electrophysiology and Pacing, Universitair Ziekenhuis Brussel-Vrije Universiteit Brussel, European Reference Networks Guard-Heart, Av. du Laerbeek 101, 1090 Jette, Brussels, Belgium; Texas Cardiac Arrhythmia Institute, St. David’s Medical Center, 3000 N Interstate Hwy 35 Suite 720, Austin, TX, USA; Department of Medicine, Montefiore Medical Center, Albert Einstein College of Medicine, Bronx, NY, USA; Kansas City Heart Rhythm Institute, 5100 W 110th St Second Floor, Overland Park, KS, USA; Texas Cardiac Arrhythmia Institute, St. David’s Medical Center, 3000 N Interstate Hwy 35 Suite 720, Austin, TX, USA; Texas Cardiac Arrhythmia Institute, St. David’s Medical Center, 3000 N Interstate Hwy 35 Suite 720, Austin, TX, USA; Texas Cardiac Arrhythmia Institute, St. David’s Medical Center, 3000 N Interstate Hwy 35 Suite 720, Austin, TX, USA; Interventional Electrophysiology, Scripps Clinic, La Jolla, CA, USA; Department of Cardiology, MetroHealth Medical Center, Case Western Reserve University School of Medicine, Cleveland, OH, USA

**Keywords:** Intracardiac echocardiography, Left atrial appendage, Stroke, Watchman, FLX, Transoesophageal echocardiography, Thromboembolism

## Abstract

**Aims:**

Left atrial appendage (LAA) imaging is critical during percutaneous occlusion procedures. 3D-intracardiac echocardiography (ICE) features direct visualization of LAA from multiple cross-sectional planes at a time. We aimed at reporting procedural success of 3D-ICE-guided LAA occlusion and the correlation between pre-procedural transoesophageal echocardiography (TEE) and intraprocedural 3D-ICE for LAA sizing.

**Methods and results:**

Among 274 patients undergoing left atrial appendage occlusion (LAAO) with a Watchman FLX, periprocedural ICE guidance was achieved via a commercially available 2D-ICE catheter (220 patients) or a novel (NUVISION™) 3D-ICE one (54 patients). Primary endpoint was a composite of procedural success and LAA sealing at follow-up TEE. Secondary endpoint was a composite of periprocedural device recapture/resizing plus presence of leaks ≥ 3 mm at follow-up TEE. 3D-ICE measurements of maximum landing zone correlated highly with pre-procedural TEE reference values [Pearson’s: 0.94; *P* < 0.001; bias: −0.06 (−2.39, 2.27)]. The agreement between 3D-ICE-based device selection and final device size was 96.3% vs. 79.1% with 2D-ICE (*P* = 0.005). The incidence of the primary endpoint was 98.1% with 3D-ICE and 97.3% with 2D-ICE (*P* = 0.99). 2D-ICE patients had a trend towards a higher incidence of periprocedural device recapture/redeployment (31.5% vs. 44.5%; *P* = 0.09). The secondary endpoint occurred in 31.5% of 3D-ICE patients vs. 45.9% of 2D-ICE ones (*P* = 0.065).

**Conclusion:**

Intracardiac echocardiography-guided LAAO showed a very high success, with no major adverse events. A very high level of agreement for LAA sizing was found between pre-procedural TEE and periprocedural 3D-ICE. 3D-ICE performed significantly better than 2D-ICE for FLX size selection and may provide better guidance during device deployment.

## Graphical Abstract

**Graphical Abstract:**
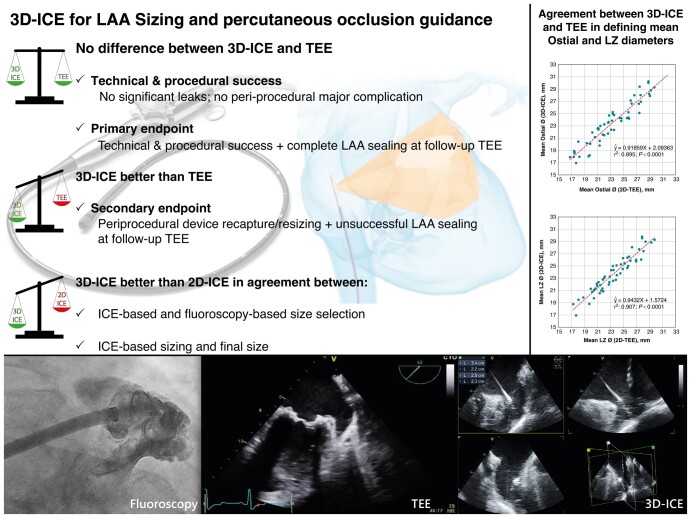

**What’s new?**

- The left atrial appendage (LAA) exhibits great variability in size, shape, number of lobes, and orientation.
- These features may limit the benefits and reliability of 2D echocardiography, since real-time visualization of the LAA is critical to optimize the safety and success of occlusion devices.
- 3D-intracardiac echocardiography (ICE)-guided LAA occlusion showed a very high technical and procedural success, with no major adverse events.
- The full potential of 3D-ICE for LAA occlusion needs to be explored in a randomized fashion, comparing different periprocedural imaging techniques.

## Introduction

Percutaneous left atrial appendage occlusion (LAAO) is routinely performed via an integrated multi-modality imaging approach based on contrast fluoroscopy and transoesophageal echocardiography (TEE).^1–6^ Periprocedural imaging is critical for success, as it allows for LAA visualization/sizing, device selection/deployment, as well as complication monitoring.

Lately, intracardiac echocardiography (ICE) has been proposed as an alternative to TEE for LAAO guidance; recent studies have not only confirmed its safety and efficacy^2,7–9^ but also reported a significant reduction in procedural time, contrast volume, and/or fluoroscopy dose^1,2^ compared to TEE-guided procedures. Two-dimensional (2D) cardiac ultrasound imaging, either transoesophageal or intracardiac, displays a certain structure along a single cross-sectional plane at a time. Therefore, a comprehensive echocardiographic assessment of complex and highly variable structures such as the LAA may require skills and experience.

The LAA exhibits great variability in size, shape, number of lobes, and orientation.^10^ These features may limit the benefits and reliability of 2D echocardiography, since real-time visualization of the LAA is critical to optimize the safety and success of occlusion devices. In this perspective, 3D echocardiography features direct visualization of the LAA complex anatomy in multiplane/multislice modes with high reproducibility, thereby overcoming the need for geometric assumptions.

Our aim was to describe the first clinical experience with a novel ICE catheter (NUVISION™ 4D ICE Catheter; Biosense Webster Inc., Irvine, CA) capable of real-time 3D imaging for LAAO procedural guidance.

## Methods

This prospective registry included 274 consecutive patients with non-valvular AF undergoing percutaneous LAAO with a Watchman FLX device (Boston Scientific Corporation, Maple Grove, MN, USA).^6,11,12^

Watchman implantation technique has been previously described elsewhere.^3,4,13–17^ All procedures were conducted under general anaesthesia; transseptal access was guided by a combination of fluoroscopy and ICE, as per operator’s preference and experience. All procedures were performed on uninterrupted oral anticoagulation.

Periprocedural ICE guidance was achieved via:

1. The novel 3D-ICE catheter (NUVISION™ 4D ICE Catheter, Biosense Webster Inc., Irvine, CA) in 54 patients (group 3D-ICE); these procedures were performed between August 2021 and March 2022 by five different highly experienced operators, or
2. A commercially available 2D-ICE catheter (10 F SoundStar™ or Acunav™ ultrasound catheter, Biosense Webster, Irvine, CA or 9 F ViewFlex Xtra ICE catheter, St. Jude Medical, St. Paul, MN) in 220 patients (group 2D-ICE); these procedures were performed between June 2021 and March 2022 by the same five operators.

The number of cases per operator included in group 2D-ICE was weighted (ratio ∼4:1) upon the contribution of the same operators in group 3D-ICE, aiming at avoiding any imbalance associated with potentially different levels of experience (see Supplementary material online, *Table S1*).

Patients were discharged after overnight observation unless they developed a periprocedural complication requiring longer hospital stay. Patients signed a written informed consent for research data collection and prior to all invasive procedures; data were collected in Institutional Review Board-approved databases.^18^ The data underlying this article will be shared on reasonable request to the corresponding author.

The aim of the study was to: (1) report technical and procedural success rate of 3D-ICE-guided LAAO; and (2) assess the correlation between pre-procedural TEE and periprocedural 3D-ICE for LAA sizing.

### Intracardiac echocardiography-guided appendage occlusion

**Figure 1:**
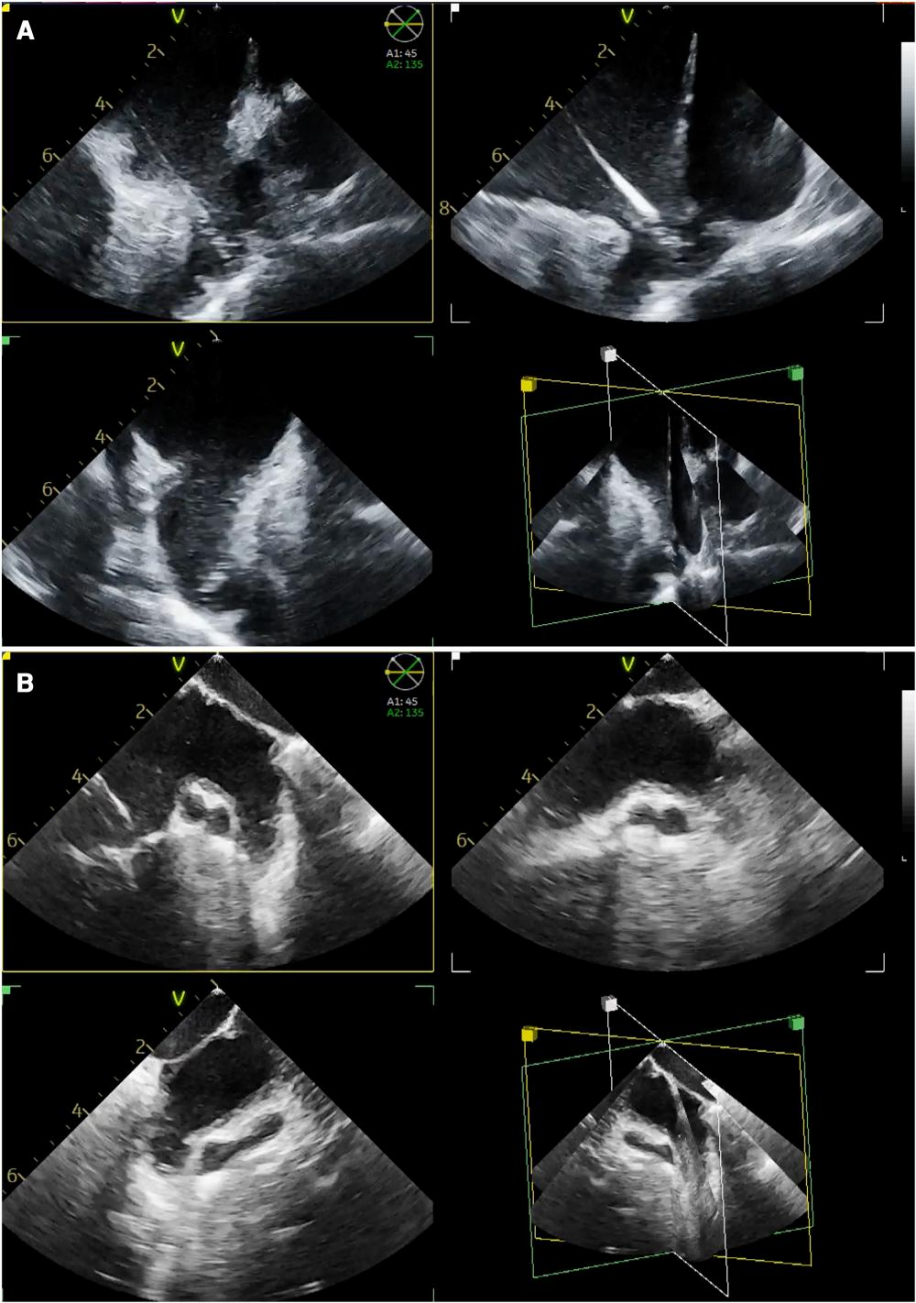
Periprocedural MPRs to assess LAA morphology and size in two patients (panels *A* and *B*). 3D-ICE MPRs (45°–90°–135°) for LAA sizing before occlusion. The Watchman sheath can be seen in the LA over pigtail wire in panel *A*. Multiplanar reconstruction allows for scanning and sizing the LAA from different angles without the need to reposition the ICE probe. ICE, intracardiac echocardiography; LAA, left atrial appendage; MPR, multiplanar reconstruction.

**Figure 2:**
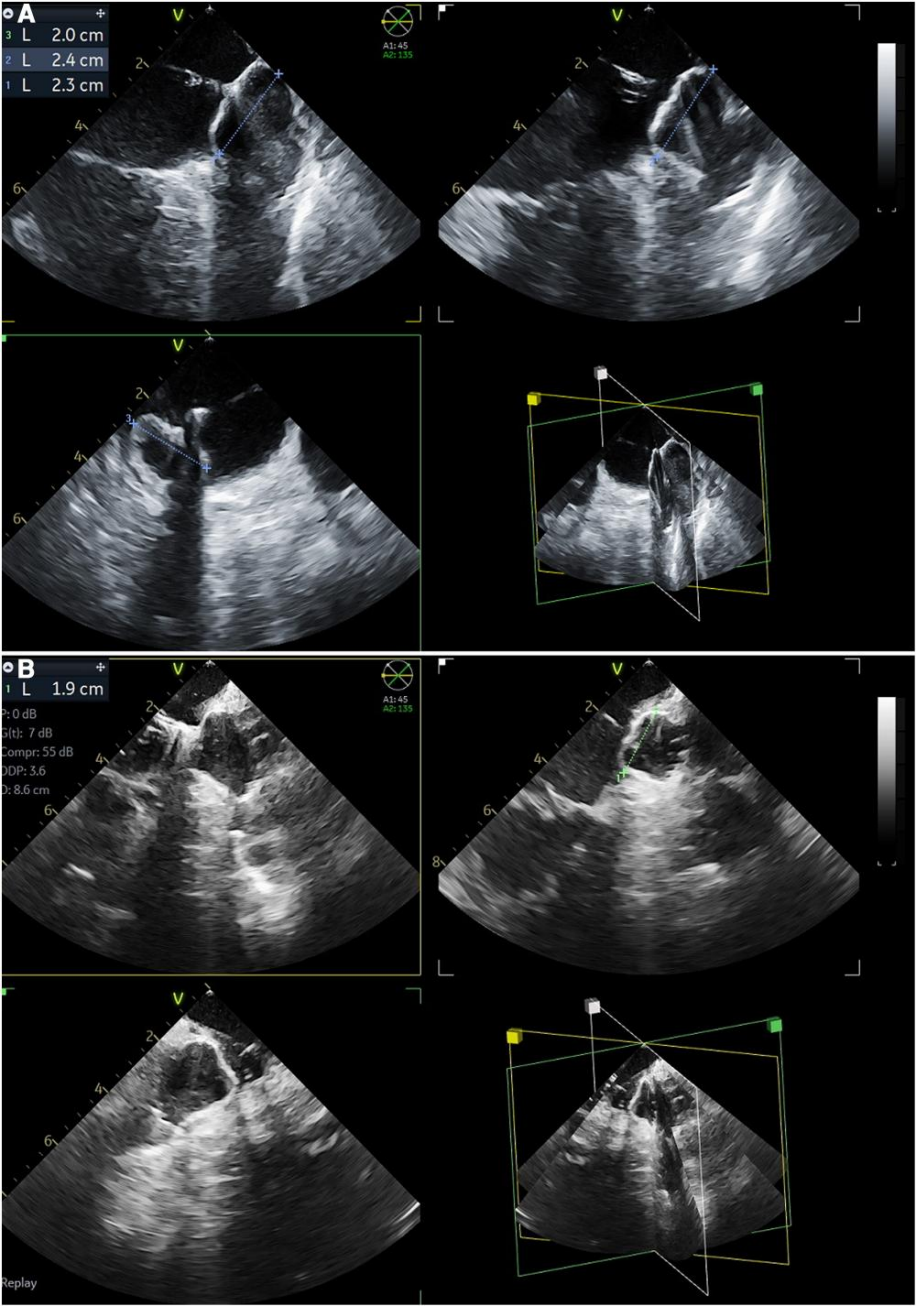
Assessment of adequate device position and compression in two patients (panels *A* and *B*). Watchman deployment and device sizing from 3D-ICE multiplanar reconstruction (45°–90°–135°) to assess stability, compression, and appendage sealing (presence of residual leaks). ICE, intracardiac echocardiography.

The ICE probe was advanced into the LA (see Supplemental methods). Device sizing (*Figures 1* and *2*) relied on multiple ICE measurements of the maximum landing zone (LZ) from several left atrial views and on fluoroscopy. The LZ was defined as the distance between the origin of the circumflex artery to a point 1 cm below the limbus of the left superior pulmonary vein (LSPV). Left atrial appendage views (*Figure 1*) were obtained from the LSPV, the left atrial body, and across the mitral valve (MV); slight manual rotations of the shaft were useful to optimize measurements and identify irregular anatomies/additional lobes.

In group 2D-ICE, the LZ was measured on multiple cross-sectional (2D) planes from each LAA view.

In group 3D-ICE, multiplanar reconstruction (MPR) was used (*Figures 1* and *2*, Supplementary material online, *Videos S1* and *S2*) to simultaneously obtain three cross-sectional planes from each LAA view. Multiplanar reconstruction cutting angles were separately rotated, aiming at optimizing the alignment of the three cross-sectional planes with the LAA axes and lobes.

Device deployment was performed under ICE guidance from the LSPV in a single 2D plane (group 2D-ICE) or in MPR mode (group 3D-ICE).

After deployment, multiple 2D views (group 2D-ICE) or MPR views (group 3D-ICE; *Figure 2*, Supplementary material online, *Video S2*) with/without colour Doppler acquired from the LSPV, the left atrial body, and across the MV were used to assess device release criteria (position, compression, presence of leaks).

### Study endpoints and definitions

Technical success was defined as successful device implantation without periprocedural evidence of leaks ≥ 3 mm on colour Doppler.

Procedural success was a composite of technical success plus absence of procedure- or device-related major complications (within 7 days after LAAO).

Primary endpoint was a composite of procedural success and complete LAA sealing at follow-up TEE. Successful sealing was defined as complete occlusion or presence of a peri-device leak < 3 mm with evidence of echo-dense thrombus beyond the occlusion device^10^ at follow-up TEE.

Secondary endpoint was a composite of periprocedural device recapture/resizing plus presence of leaks ≥ 3 mm at follow-up TEE.

Device recapture was defined as the need for partial or complete FLX recapture into the delivery system due to suboptimal device deployment/LAA occlusion.

Device resizing was defined as the need for a device size change due to suboptimal device deployment/LAA occlusion with an initial device size. Multiple recaptures or device size changes in the same patient were counted as a single event.

The agreement between ICE and fluoroscopy was defined as the % of cases where the two techniques predicted the same device size according to the manufacturer sizing chart and with an estimated compression rate ≥ 15%.

The agreement between ICE and final size was defined as the % of cases where ICE measurements predicted the same device size as that finally deployed into the LAA.

### 3D-intracardiac echocardiography vs. transoesophageal echocardiography measurements of the left atrial appendage

All patients undergoing 3D-ICE-guided LAAO had an elective 2D-TEE done within 60 days before the occlusion procedure (see Supplementary material online, *Figure S1*). At pre-procedural TEE, the LAA was imaged in four different views (0–20°, 45–60°, 90°, and 120–135°). 2D-TEE and 3D-ICE images were digitally stored for off-line analysis. Four cross-sectional TEE images (one in each of the four views) and two MPR views from 3D-ICE (one from the LSPV and one from across the MV) were anonymized and submitted unmatched and in random order to a cardiologist (M.M.) experienced in TEE and ICE imaging. Off-line measurements were performed via Meander software v3.1.1 (Peacock media, Saratoga Springs, NY, USA). The reviewer provided two measurements of the ostial and LZ diameters per image. We compared mean and maximum ostial diameters and mean and maximum LZ diameters obtained from same patient’s TEE and 3D-ICE; these measurements were used to assess the degree of correlation and agreement between the two imaging techniques. Mean and maximum ostial/LZ diameters were calculated as the mean of 8 measurements from 4 cross-sectional TEE views or the mean of 12 measurements from 2 MPR 3D-ICE views per patient.

### Statistical analysis

Statistical analysis was performed via IBM SPSS Statistics 25.0 (IBM SPSS Inc., Chicago, IL, USA). Categorical and continuous variables were presented using absolute values (percentage) and as mean ± standard deviation [median and inter-quartile range (IQR) for non-normal data], respectively. Statistical significance was defined as *P* < 0.05. Further details are reported in the Supplemental methods.

## Results

Overall, 274 consecutive AF patients (mean age: 74.8 ± 8.8 years; 64.2% males) undergoing an attempted LAAO procedure with a Watchman FLX were included. The procedure was conducted under 3D-ICE guidance in 54 patients (group 3D-ICE) or 2D-ICE in 220 others (group 2D-ICE). Baseline characteristics are reported in *Table 1*.

**Table 1:** Baseline characteristics

Demographics	3D-ICE (*n* = 54)	2D-ICE (*n* = 220)	*P*-value
Age, yrs	74.9 ± 8.9	74.8 ± 8.9	0.91
Female	18 (33.3)	80 (36.4)	0.75
BMI, kg/m^2^	28.8 ± 6.3	28.7 ± 5.9	0.91
CHA_2_DS_2_-VASc score	4.6 ± 1.2	4.4 ± 1.5	0.43
HAS-BLED score	3.0 ± 1.0	3.1 ± 1.3	0.60
CHF	26 (48.1)	91 (41.4)	0.44
Hypertension	51 (94.4)	193 (87.7)	0.22
Diabetes mellitus	22 (40.7)	77 (35.0)	0.53
Hx. of stroke/TIA/SE	20 (37.0)	98 (44.5)	0.36
Vascular disease	25 (46.3)	93 (42.3)	0.65
CAD	15 (27.8)	49 (22.3)	0.47
Abnormal renal function	3 (5.6)	16 (7.2)	0.77
Hx. of major bleeding	29 (53.7)	129 (58.9)	0.54
Drug interactions	20 (37.0)	99 (45.0)	0.36
Alcohol	4 (7.4)	21 (9.5)	0.79
LVEF [range]	52 ± 12 [25–65]	51 ± 12 [20–65]	0.73

BMI, body mass index; CAD, coronary artery disease; CHF, congestive heart failure; ICE, intracardiac echocardiography; INR, international normalized ratio; LVEF, left ventricular ejection fraction; SE, systemic embolism; TIA, transient ischaemic attack.

### 3D-intracardiac echocardiography vs. 2D-intracardiac echocardiography-guided left atrial appendage occlusion: probe access into left atrium and manoeuvrability

The 3D-ICE probe successfully accessed the LA in all 54 group 3D-ICE patients. Left atrium access was achieved by directly advancing the ICE probe through the TS point (85.2%, 46 patients) or through a 10 F TS sheath exchanged over the wire after several attempts to directly cross the septum with the ICE probe were unsuccessful (14.8%, 8 patients).

The 2D-ICE probe successfully accessed the LA in all 220 group 2D-ICE patients. Left atrium access was achieved by directly advancing the ICE probe through the second TS point (88.6%, 195 patients) or through a 10 F TS sheath exchanged over the wire after several attempts to directly cross the septum were unsuccessful (11.4%, 25 patients).

3D-ICE manoeuvrability into the LA was rated as ‘very good’ (75.9%, 41 patients) or ‘good’ (13.0%, 7 patients) in 88.9% of cases (*n* = 48). The rating was ‘neutral’ (5.55%, 3 patients) or ‘poor’ (5.55%, 3 patients) in the remaining six cases, all of whom had required a 10 F sheath to facilitate the 3D-ICE tip access into the LA (see Supplementary material online, *Figure S1*). The new rotation knob feature was rated as ‘very good’ or ‘good’ in 46 (85.2%) cases, with satisfaction increasing from 75% for the initial 20 cases (first 4 cases per operator) to 91.2% for the following 34 patients (see Supplementary material online, *Figure S2*). Similarly, a trend towards shorter procedural times was observed from the same initial 20 cases (42.7 ± 7.7 min) to the remaining 34 patients (39.6 ± 6.3 min; *P* = 0.10).

### 3D-intracardiac echocardiography vs. 2D-intracardiac echocardiography-guided left atrial appendage occlusion: device sizing

All procedural details are reported in *Table 2*. The agreement between 3D-ICE and fluoroscopy for device sizing was 94.4% (*n* = 51); in the other three (5.6%) patients, 3D-ICE indicated one size larger (2 pts) or smaller (1 pt) than that selected with fluoroscopy. The agreement between 3D-ICE-based device selection and final device size was 96.3% (*n* = 52). Device recapture was required in 17 (31.5%) cases, whereas no resizing was necessary.

**Table 2:** Procedural and follow-up details

Procedural and follow-up details	3D-ICE (*n* = 54)	2D-ICE (*n* = 220)	*P*-value
Watchman FLX size			
20 mm	4 (7.4)	17 (10.5)	0.61
24 mm	12 (22.2)	39 (24.1)	0.85
27 mm	14 (26.0)	36 (22.2)	0.71
31 mm	18 (33.3)	41 (25.3)	0.29
35 mm	6 (11.1)	29 (17.9)	0.29
Device recapture	17 (31.5)	98 (44.5)	0.09
Device resizing	0 (0.0)	4 (1.8)	0.56
Agreement ICE-fluoroscopy	51 (94.4)	174 (79.1)	**0**.**008**
Agreement ICE-final size	52 (96.3)	177 (80.4)	**0**.**005**
Fluoroscopy time, min	7.6 ± 2.8	8.4 ± 3.8	0.11
Contrast volume, mL	63 ± 26	69 ± 23	0.14
Procedural duration, min	40.1 ± 8.4	41.8 ± 8.8	0.17
Hospital length of stay, day	1 [1–1]	1 [1–1]	0.84
Leaks ≥ 3 mm at follow-up	1 (1.9)	6 (2.7)	0.99
Technical success^a^	54 (100)	220 (100)	1
Procedural success^b^	54 (100)	220 (100)	1
Primary endpoint^c^	53 (98.1)	214 (97.3)	1
Secondary endpoint^d^	17 (31.5)	101 (45.9)	0.06

ICE, intracardiac echocardiography; MI, myocardial infarction; TIA, transient ischaemic attack. Statistically significant values are reported in bold.

^a^Successful device implantation without periprocedural evidence of leaks ≥ 3 mm on colour Doppler.

^b^Composite of technical success plus absence of procedure- or device-related major complications (within 7 days after LAAO).

^c^Composite of procedural success and complete LAA sealing at follow-up TEE.

^d^Composite of periprocedural device recapture/resizing plus unsuccessful LAA sealing (leaks ≥ 3 mm) at follow-up TEE.

The agreement between 2D-ICE and fluoroscopy for device sizing was 79.1% (*n* = 174). The device indicated by 2D-ICE measurements was one size smaller than that suggested by fluoroscopy in 91.3% (*n* = 42) of cases and larger in the remaining 8.7% (*n* = 4). The agreement between 2D-ICE-based device selection and final device size was 80.4% (*n* = 177).

2D-ICE patients had a trend towards a higher incidence of recapture(s)/redeployment(s) to optimize device placement and LAA sealing (31.5% vs. 44.5%; *P* = 0.09).

The agreement between ICE-based and fluoroscopy-based size selection (group 3D-ICE: 94.4% vs. group 2D-ICE: 79.1%, *P* = 0.008) and between ICE-based sizing and final size (group 3D-ICE: 96.3% vs. group 2D-ICE: 80.4%, *P* = 0.005) was significantly higher with 3D-ICE.

### 3D-intracardiac echocardiography vs. 2D-intracardiac echocardiography-guided left atrial appendage occlusion: technical and procedural success

Technical and procedural success was achieved in all 274 patients; all final devices met release criteria (*Table 2*). No major procedure- and device-related adverse events were documented (*Table 3*); two (0.9%) patients in group 2D-ICE developed a right groin haematoma (contralateral to venous access side for the ICE catheter).

**Table 3:** Periprocedural complications

Periprocedural complications	3D-ICE (*n* = 54)	2D-ICE (*n* = 220)	*P*-value
Death	0	0	—
Stroke/TIA	0	0	—
Air embolism	0	0	—
MI	0	0	—
Major bleeding	0	0	—
Device embolization	0	0	—
Pericardial effusion	0	0	—
Surgery	0	0	—
Percutaneous drainage	0^a^	0	—
No intervention	0	0	—
Vascular complication	0	0	—
Retroperitoneal haematoma	0	0	—
Groin haematoma	0	2 (0.9)	0.99
Major adverse events	0	0	—
Overall adverse events	0	2 (0.9)	0.99

ICE, intracardiac echocardiography; MI, myocardial infarction; TIA, transient ischaemic attack.

^a^A 73-year-old asymptomatic female patient was scheduled for Watchman implantation the day after a left-sided ablation procedure. Intracardiac echocardiography showed a newly formed pericardial effusion. A percutaneous drainage was placed and the effusion evacuated before Watchman implantation.

### 3D-intracardiac echocardiography vs. 2D-intracardiac echocardiography-guided left atrial appendage occlusion: study endpoints

Follow-up TEE was performed after a mean of 56 ± 9 days, revealing no leaks ≥ 5 mm and seven (2.6%) patients with a 3–4 mm leak [group 3D-ICE: 1.9% (*n* = 1) vs. group 2D-ICE: 2.7% (*n* = 6); *P* = 0.99].

The incidence of the primary endpoint (procedural success and complete appendage sealing) was 98.1% (*n* = 53) in group 3D-ICE and 97.3% (*n* = 214) in group 2D-ICE (*P* = 0.99).

The incidence of the secondary endpoint (device recapture/resizing or peri-device leaks ≥ 3 mm) was 31.5% (*n* = 17) among 3D-ICE patients vs. 45.9% (*n* = 101) among 2D-ICE ones (risk ratio: 0.69; 95% confidence intervals: 0.45–1.04; *P*-value: 0.065). Of note, one 3D-ICE and three 2D-ICE patients had both a periprocedural device recapture and a leak ≥ 3 mm at follow-up and these events were counted as one secondary endpoint event.

### 3D-intracardiac echocardiography vs. transoesophageal echocardiography measurements of the left atrial appendage

**Figure 3:**
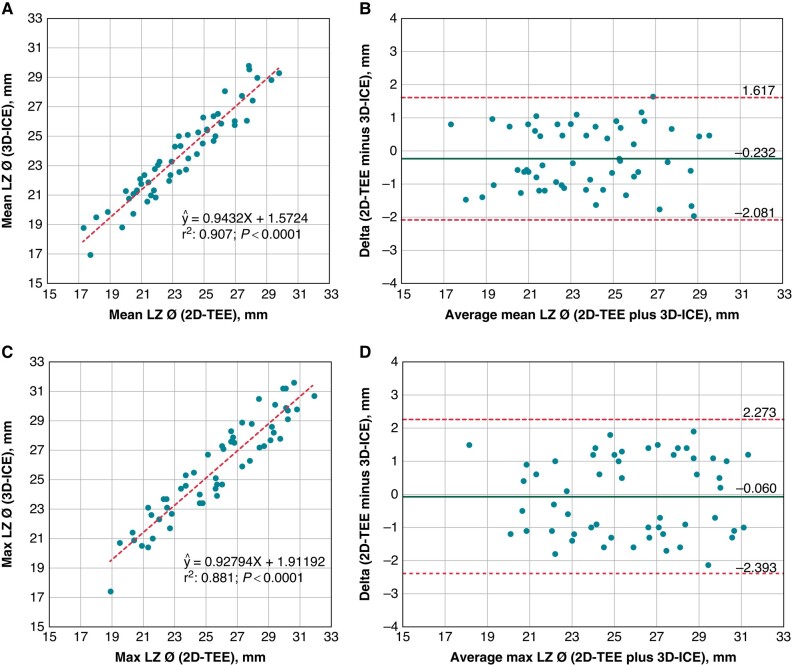
Scatterplots of linear regression analysis (panels *A* and *C*) and Bland–Altman plots comparing mean landing zone diameters (panels *A* and *B*) and maximum landing zone diameters (panels *C* and *D*) measured via 3D-ICE and 2D-TEE. Ø, diameter; ICE, intracardiac echocardiography; LZ, landing zone; TEE, transoesophageal echocardiography.

The distribution of LAA morphologies among group 3D-ICE patients (*n* = 54) was: chicken wing: 44.4% (*n* = 24); cactus: 27.8% (*n* = 15); windsock: 20.4% (*n* = 11); cauliflower: 7.4% (*n* = 4); a dual lobe anatomy was documented in 57.4% (*n* = 31) patients. All patients had a pre-procedural TEE performed 41 ± 9 days before the LAAO procedure. Periprocedural 3D-ICE measurements of the ostium and LZ were consistent with pre-procedural TEE. Mean (see Supplementary material online, *Figure S3A* and *B*) and maximum (see Supplementary material online, *Figure S3C* and *D*) ostial diameters were consistent between TEE and 3D-ICE, resulting in a Pearson’s correlation coefficient of 0.95 (*P* < 0.001) and 0.94 (*P* < 0.001), respectively. Mean LZ was 23.51 ± 3.06 mm with TEE vs. 23.74 ± 3.04 mm with 3D-ICE (Pearson’s: 0.95; *P* < 0.001), with a small negative bias (−0.232) and narrow limits of agreement (−2.08, 1.62) (*Figure 3A* and *B*).

Similarly, 3D-ICE measurements of maximum LZ correlated highly with 2D-TEE reference values [Pearson’s: 0.94; *P* < 0.001; bias: −0.06 (−2.39, 2.27); *Figure 3C* and *D*].

## Discussion

Herein we report the first multicentre experience with 3D-ICE for LAAO with a Watchman FLX device. Our study aimed at assessing safety and feasibility of 3D-ICE for LAAO procedures and validating its reliability for periprocedural LAA sizing compared to pre-procedural TEE.

The main findings of our study are the following:

1. 3D-ICE-guided LAAO was safe and feasible. Technical and procedural success was achieved in 100% of 3D-ICE-guided procedures, with no major adverse events.
2. The agreement between ICE-based and fluoroscopy-based device sizing and between ICE-based device selection and final device size was significantly higher with 3D-ICE.
3. The incidence of the primary endpoint (procedural success and complete appendage sealing) did not differ between imaging groups.
4. The number of patients requiring periprocedural device recapture to optimize placement and sealing was higher with 2D-ICE; however, the difference was not significant (*P* = 0.09).
5. A trend towards a higher incidence of the secondary endpoint of device recapture/resizing or peri-device leaks ≥ 3 mm (*P* = 0.065) was observed among 2D-ICE patients.
6. A high level of agreement for LAA sizing was found between pre-procedural TEE and periprocedural 3D-ICE.

### Procedural details of 3D-intracardiac echocardiography-guided left atrial appendage occlusion

An accurate and reliable determination of appendage geometry has important clinical implications for percutaneous occlusion procedures.^19^ Specifically, a comprehensive assessment of LAA dimensions and anatomical features, as well as its relationships with neighbouring structures, correlates with procedural safety and successful sealing.

In combination with contrast fluoroscopy, 2D echocardiography, either transoesophageal or intracardiac, is the imaging modality of choice to guide device selection/deployment and confirm adequate appendage sealing.

Recent comparative studies have shown that 2D-ICE from the LA is as safe and effective as TEE^1,2,20,21^; also, intracardiac ultrasound was shown to contribute to shorter procedural and turnover times. However, these advantages may manifest after an adequate learning curve due to an increased level of complexity resulting from ICE catheter manipulation in the LA.

In our experience on 274 LAAO patients, both 2D- and 3D-ICE safely and effectively guided appendage occlusion, contributing to 100% acute success, no periprocedural complications, and a very low rate of significant leaks.

2D-ICE patients showed a trend towards a higher need for periprocedural device recapture/repositioning to optimize sealing and avoid uncovered proximal pouches/lobes. This finding was the main determinant for the higher, though not significant, incidence of the secondary endpoint of recapture, resizing, and suboptimal LAA sealing (leaks ≥ 3 mm) at follow-up. We hypothesize that this endpoint did not reach statistical significance because of the high level of experience of the operators involved in our study.

However, 3D-ICE performed significantly better than 2D-ICE for device size selection; specifically, the level of agreement between ICE and fluoroscopy, as well as between ICE and final device size was higher with 3D-ICE (*P* = 0.008 and 0.005, respectively). This observation is key and, unlike 2D-ICE, may support the adoption of the 3D technology for ICE-guided-only LAA occlusion procedures. In this perspective, 3D-ICE features simultaneous visualization of the LAA from different cross-sectional planes; therefore, less experienced operators are more likely to benefit from a better understanding of appendage geometry and guidance for sizing and deployment.^22^ The result might be a simplified, safer, and optimized LAAO workflow. In this perspective, 3D-ICE, in combination with MPR, may limit the need for continuous adjustments of the position of the ICE probe, thereby providing uninterrupted guidance during device deployment and reducing operator dependency. Additionally, less device recaptures/redeployments may further decrease the risk of periprocedural complications.^23–25^

### Technical considerations and implications on left atrial appendage occlusion workflow

**Figure 4:**
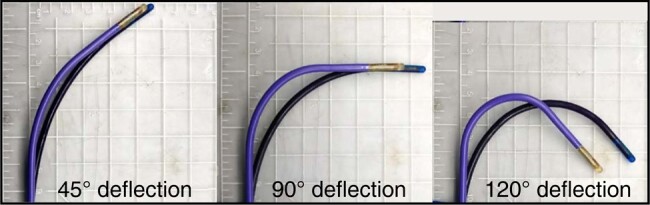
Deflection comparison between ACUNAV™ (black, Biosense Webster, Irvine, CA) and NUVISION™ (purple, Biosense Webster, Irvine, CA) ultrasound catheters. Deflections were conducted at room temperature. Modified and reproduced with permission from Biosense Webster, Irvine, CA.

The NUVISION™ 4D ICE is a novel 10 Fr catheter with fully sampled matrix phased-array transducer capable of real-time 3D imaging. A unique feature of this device is the presence of a dedicated knob, which allows independent 360° rotation of the tip. The rotation knob provides a critical advantage for LAAO procedure. Once a stable position is achieved in the LA, the combination of tip rotations through the knob and multiplanar visualization of the appendage from adjustable views can limit the need for catheter manipulation and help optimize LAA geometrical assessment. In our experience, operators’ satisfaction rose from 75% for the initial 20 cases to 91.2% for the following 34 patients, as a result of the better familiarity with the rotation knob. Although the rotation knob can be remarkably helpful for many interventional procedures, it functions best when returned to its neutral or ‘home’ position so that it mimics the expected fixed antero-posterior transducer position of 2D catheters when anteflexed to cross the septum. As the reach and shape of the deflected NUVISION™ are slightly different from other commercially available catheters (*Figure 4*), the combination of deflection and knob rotation away from the neutral position can limit the control of the tip and result in a more difficult transseptal crossing. Of note, the increasing manoeuvrability and familiarity with the rotation knob were associated with a trend towards shorter procedural times.

Finally, a critical finding of our study is the high level of agreement observed between pre-procedural TEE and periprocedural 3D-ICE for LAA ostial and LZ measurements. It is known that an accurate LAA sizing is pivotal for successful occlusion, especially in patients with high ostial eccentricity and multiple lobes. In our study, TEE and 3D-ICE images were anonymized and submitted unmatched and in random order for revision to avoid any expectation bias. Previous studies have shown the safety and feasibility of performing AF ablation in patients on uninterrupted DOACs without pre-procedural TEE. These previous observations, in combination with the high level of agreement between pre-procedural TEE and periprocedural 3D-ICE for LAA ostial and LZ sizing, may justify future studies investigating the feasibility of 3D-ICE-guided LAAO without screening TEE. This strategy may have important clinical and economic implications.

## Limitations

Our study has several limitations that need to be acknowledged. (1) The lack of randomization may limit the interpretation of these data and introduce all the limitations and biases related to its design. (2) Left atrial appendage occlusion procedures were performed by five highly experienced operators at three centres, and results may not be generalizable to less experienced operators. (3) Caution should be applied when extrapolating our observations to different occlusion devices or 3D-ICE catheters. (4) Our study focused exclusively on the safety and efficacy of ICE-guided LAAO and did not address any issues related to the efficacy of occlusion devices for stroke prevention and bleeding risk in the short- and long-term.

## Conclusions

3D-ICE-guided LAAO showed a very high technical and procedural success, with no major adverse events. A high level of agreement for LAA sizing was found between pre-procedural TEE and periprocedural 3D-ICE. Compared to 2D-intracardiac ultrasound, 3D-ICE performed significantly better than 2D-ICE for device sizing and showed a trend towards a lower incidence of the composite endpoint of periprocedural device recapture/resizing and significant peri-device leaks at follow-up.

## Supplementary Material

euae010_Supplementary_Data

## Data Availability

All relevant data are within the manuscript and its supporting information files.

## References

[1] Gianni C , Horton RP, Della Rocca DG, Mohanty S, Al-Ahmad A, Bassiouny MA et al Intracardiac echocardiography- versus transesophageal echocardiography-guided left atrial appendage occlusion with Watchman FLX. Cardiovasc Electrophysiol 2021;32:2781–4. 10.1111/jce.15220 34411376

[2] Korsholm K , Jensen JM, Nielsen-Kudsk JE. Intracardiac echocardiography from the left atrium for procedural guidance of transcatheter left atrial appendage occlusion. JACC: Cardiovasc Interv 2017;10:2198–206.28866042

[3] Kleinecke C , Yu J, Neef P, Buffle E, De Marchi S, Fuerholz M et al Clinical outcomes of Watchman vs. Amplatzer occluders for left atrial appendage closure (WATCH at LAAC). Europace 2020;22:916–23. 32003774 10.1093/europace/euaa001

[4] Messele LF , Khan MZ, Darden D, Agarwal S, Krishan S, Pasupula DK et al Outcomes of percutaneous left atrial appendage occlusion device implantation in atrial fibrillation patients based on underlying stroke risk. Europace 2023;25:1415–22. 36881781 10.1093/europace/euad049 PMC10105852

[5] Mir T , Rawasia WF, Uddin M, Sheikh M, Munir MB, Balla S. Left atrial appendage closure device outcomes among cirrhosis patients with atrial fibrillation: a United States National Cohort Study. Europace 2023;25:1408–14. 36857522 10.1093/europace/euad004 PMC10105884

[6] Betts TR , Grygier M, Nielsen Kudsk JE, Schmitz T, Sandri M, Casu G et al Real-world clinical outcomes with a next-generation left atrial appendage closure device: the FLXibility post-approval study. Europace 2023;25:914–21. 36734247 10.1093/europace/euac270 PMC10062328

[7] Berti S , Paradossi U, Meucci F, Trianni G, Tzikas A, Rezzaghi M et al Periprocedural intracardiac echocardiography for left atrial appendage closure. JACC: Cardiovasc Interv 2014;7:1036–44. 25234677 10.1016/j.jcin.2014.04.014

[8] Matsuo Y , Neuzil P, Petru J, Chovanec M, Janotka M, Choudry S et al Left atrial appendage closure under intracardiac echocardiographic guidance: feasibility and comparison with transesophageal echocardiography. J Am Heart Assoc 2016;5:e003695. 27680664 10.1161/JAHA.116.003695 PMC5121476

[9] Hemam ME , Kuroki K, Schurmann PA, Dave AS, Rodríguez DA, Sáenz LC et al Left atrial appendage closure with the Watchman device using intracardiac vs transesophageal echocardiography: procedural and cost considerations. Heart Rhythm 2019;16:334–42. 30827462 10.1016/j.hrthm.2018.12.013 PMC6400300

[10] Ayhan H , Mohanty S, Gedikli Ö, Trivedi C, Canpolat U, Tapia AC et al A simple method to detect leaks after left atrial appendage occlusion with Watchman. J Cardiovasc Electrophysiol 2020;31:2338–43. 32596864 10.1111/jce.14641

[11] Della Rocca DG , Magnocavallo M, Gianni C, Mohanty S, Natale VN, Al-Ahmad A et al Procedural and short-term follow-up outcomes of Amplatzer amulet occluder versus Watchman FLX device: a meta-analysis. Heart Rhythm 2022;19: 1017–8. 35158089 10.1016/j.hrthm.2022.02.007

[12] Romero J , Gabr M, Patel K, Briceno D, Diaz JC, Alviz I et al Efficacy and safety of left atrial appendage electrical isolation during catheter ablation of atrial fibrillation: an updated meta-analysis. Europace 2021;23:226–37. 33324978 10.1093/europace/euaa266

[13] Gianni C , Anannab A, Sahore Salwan A, Della Rocca DG, Natale A, Horton RP. Closure of the left atrial appendage using percutaneous transcatheter occlusion devices. J Cardiovasc Electrophysiol 2020;31:2179–86. 32249473 10.1111/jce.14471

[14] Della Rocca DG , Magnocavallo M, Di Biase L, Mohanty S, Trivedi C, Tarantino N et al Half-dose direct oral anticoagulation versus standard antithrombotic therapy after left atrial appendage occlusion. JACC: Cardiovasc Interv 2021;14:2353–64. 34656496 10.1016/j.jcin.2021.07.031

[15] Kar S , Doshi SK, Sadhu A, Horton R, Osorio J, Ellis C et al Primary outcome evaluation of a next-generation left atrial appendage closure device: results from the PINNACLE FLX trial. Circulation 2021;143:1754–62. 33820423 10.1161/CIRCULATIONAHA.120.050117

[16] Pasupula DK , Siddappa Malleshappa SK, Munir MB, Bhat AG, Anandaraj A, Jakkoju A et al Combined atrial fibrillation ablation and left atrial appendage occlusion procedure in the United States: a propensity score matched analysis from 2016–2019 national readmission database. Europace 2023;25:390–9. 36350997 10.1093/europace/euac181 PMC9935040

[17] Ledwoch J , Sievert K, Boersma LVA, Bergmann MW, Ince H, Kische S et al Initial and long-term antithrombotic therapy after left atrial appendage closure with the WATCHMAN. Europace 2020;22:1036–43. 32464648 10.1093/europace/euaa074

[18] Della Rocca DG , Di Biase L, Mohanty S, Trivedi C, Gianni C, Romero J et al Targeting non-pulmonary vein triggers in persistent atrial fibrillation: results from a prospective, multicentre, observational registry. Europace 2021;23:1939–49. 34417816 10.1093/europace/euab161

[19] Ellis CR , Jackson GG, Kanagasundram AN, Mansour M, Sutton B, Houle VM et al Left atrial appendage closure in patients with prohibitive anatomy: insights from PINNACLE FLX. Heart Rhythm 2021;18:1153–61. 33957090 10.1016/j.hrthm.2021.02.022

[20] Masson J-B , Kouz R, Riahi M, Nguyen Thanh HK, Potvin J, Naim C et al Transcatheter left atrial appendage closure using intracardiac echocardiographic guidance from the left atrium. Can J Cardiol 2015;31:1497.e7–e14. 10.1016/j.cjca.2015.04.031 26255216

[21] Della Rocca DG , Magnocavallo M, Van Niekerk CJ, Gilhofer T, Ha G, D’Ambrosio G et al Prognostic value of chronic kidney disease in patients undergoing left atrial appendage occlusion. Europace 2023;25:euad315. 37889200 10.1093/europace/euad315 PMC10653166

[22] Della Rocca DG , Gianni C, Magnocavallo M, Mohanty S, Al-Ahmad A, Tschopp DR et al 3-Dimensional intracardiac echocardiography-guided percutaneous closure of a residual leak via radiofrequency applications after LAAO. JACC: Clin Electrophysiol 2022;8:1609–12. 36543517 10.1016/j.jacep.2022.09.019

[23] Ellis CR , Metawee M, Piana RN, Bennett JM, Pretorius M, Deegan RJ. Feasibility of left atrial appendage device closure following chronically failed surgical ligation. Heart Rhythm 2019;16:12–7. 30012348 10.1016/j.hrthm.2018.07.017

[24] Della Rocca DG , Horton RP, Di Biase L, Bassiouny M, Al-Ahmad A, Mohanty S et al First experience of transcatheter leak occlusion with detachable coils following left atrial appendage closure. JACC: Cardiovasc Interv 2020;13:306–19. 31954677 10.1016/j.jcin.2019.10.022

[25] Charate R , Ahmed A, Della Rocca DG, Bloom S, Garg J, Pothineni NVK et al Evaluation of multimodality LAA leak closure methods following incomplete occlusion. JACC: Cardiovasc Interv 2022;15:2158–70. 36357020 10.1016/j.jcin.2022.08.034

