# Supplementary material for: Three-dimensional intracardiac echocardiography for left atrial appendage sizing and percutaneous occlusion guidance
Source: Europace. 2024 Jan 16;26(1):euae010. doi: 10.1093/europace/euae010 (PMC10823354; doi:10.1093/europace/euae010)
Supplement: euae010_Supplementary_Data [file euae010_supplementary_data.zip › Supplemental material clean.docx]

**SUPPLEMENTAL MATERIALS**

**Three-Dimensional Intracardiac Echocardiography for Left Atrial Appendage Sizing and Percutaneous Occlusion Guidance**

*Della Rocca, DG, Natale A, et al.*

**Supplemental Methods.**

*ICE-Guided Appendage Occlusion.*

**Supplemental Figures.**

*Supplemental Figure 1.*

*Supplemental Figure 2.*

**Supplemental Table.**

*Supplemental Table 1.*

**Supplemental Methods.**

*ICE-Guided Appendage Occlusion.*

After the first transseptal (TS) access for the Watchman delivery system was achieved, a second puncture was performed to allow positioning of the ICE catheter into the left atrium (LA). A guidewire was advanced through an 8.5F sheath (MERIT HeartSpan or Abbott SL0) into the left superior pulmonary vein (LSPV); the sheath was then retracted into the right atrium and the wire used as a reference to visually identify the second TS point on ICE and access the LA. If any attempts to cross the septum directly with the ICE catheter were unsuccessful, the 8.5F sheath was exchanged over the wire with a 10F sheath (Abbott SL1) that was used to advance the ultrasound catheter into the LA.

*Statistical Analysis.*

The relationship between TEE and 3D-ICE measurements was assessed using linear regression analysis; correlations were examined with Pearson’s coefficient. The agreement between TEE and 3D-ICE measurements was evaluated using Bland-Altman analysis by calculating the mean differences and the limits of agreement (±1.96 standard deviation around the mean difference). For procedures performed under 3D-ICE guidance, a 5-point Likert scale was used to rate the operator satisfaction with catheter maneuverability into the LA and LAA image quality (very poor, poor, neutral, good, very good).

**Figure Legend.**

**Supplemental Figure 1. Back-to-back Comparison among 3D-ICE-based Multiplanar Reconstruction Measurements (Panel A) and Preprocedural TEE Images (Panel B).** *ICE: intracardiac echocardiography; TEE: transesophageal echocardiography.*

**Supplemental Figure 2. Operators’ Satisfaction with 3D-ICE Catheter Maneuverability (Panel A) and Rotation (Panel B).** Satisfaction was assessed via a 5-point Likert scale (very poor, poor, neutral, good, very good). Each panel reports the ratings for the first 20 cases (right columns) vs overall procedures (left column). *ICE: intracardiac echocardiography.*

**Supplemental Figure 3. Scatterplots of Linear Regression Analysis (Panels A and C) and Bland-Altman Plots Comparing Mean Ostial Diameters (Panels A and B) and Maximum Ostial Diameters (Panels C and D) Measured via 3D-ICE and 2D-TEE.** *Ø: diameter; ICE: intracardiac echocardiography; TEE: transesophageal echocardiography.*

**Supplemental Table 1. Number of Operators and Distribution of Procedures among the three Institutions.** *ICE: intracardiac echocardiography.*

| Institution | Operators, n | 2D ICE  n, (%) | 3D ICE  n, (%) |
| --- | --- | --- | --- |
| Inst. 1 | 3 | 166 (75.4) | 39 (72.2) |
| Inst. 2 | 1 | 29 (13.2) | 8 (14.8) |
| Inst. 3 | 1 | 25 (11.4) | 7 (13) |
