# Supplementary figures and images for: Three-dimensional intracardiac echocardiography for left atrial appendage sizing and percutaneous occlusion guidance
Source: Europace. 2024 Jan 16;26(1):euae010. doi: 10.1093/europace/euae010 (PMC10823354; doi:10.1093/europace/euae010)

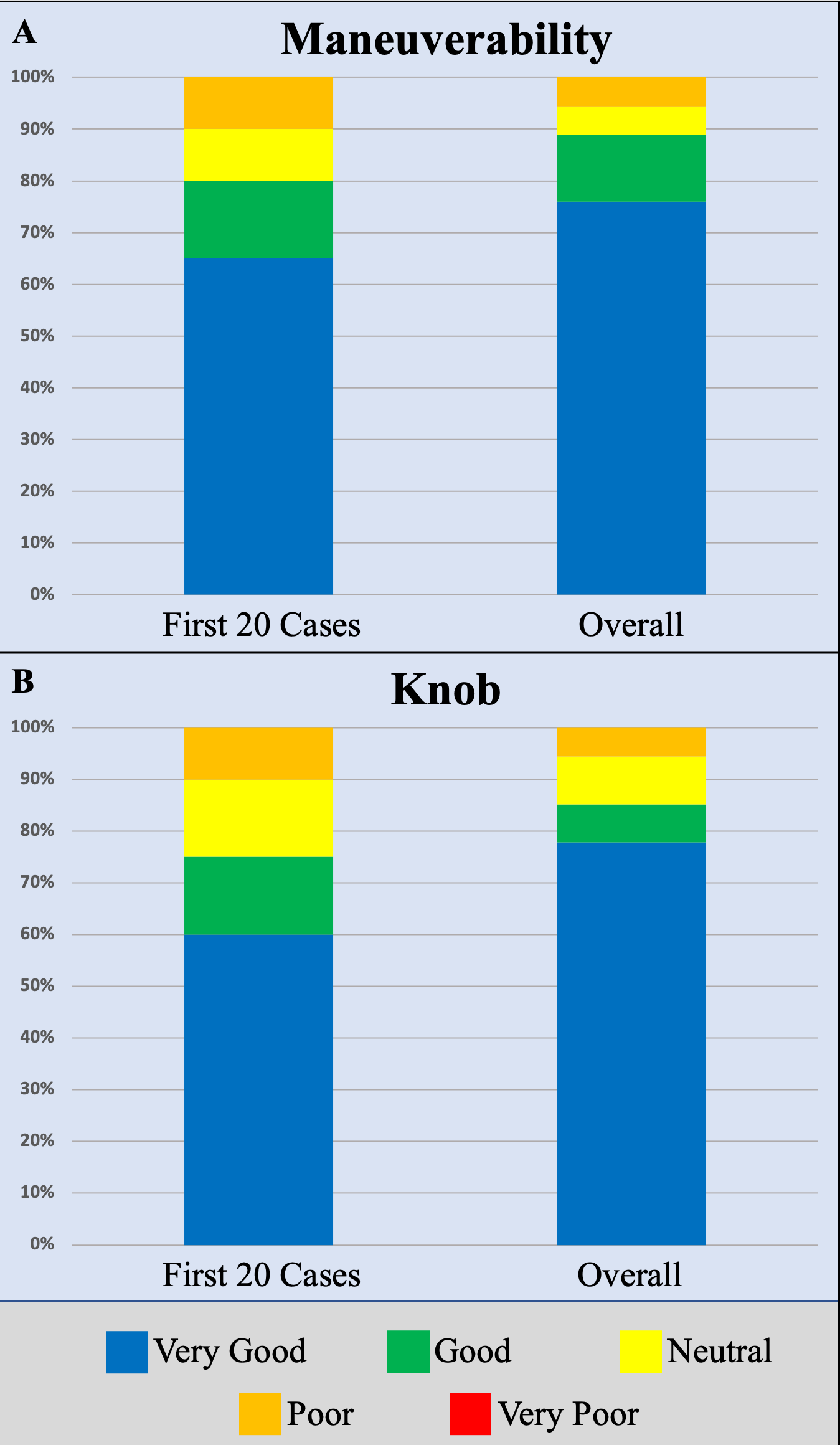

Supplement: euae010_Supplementary_Data [file euae010_supplementary_data.zip › Supplemental Fig.2.tiff]

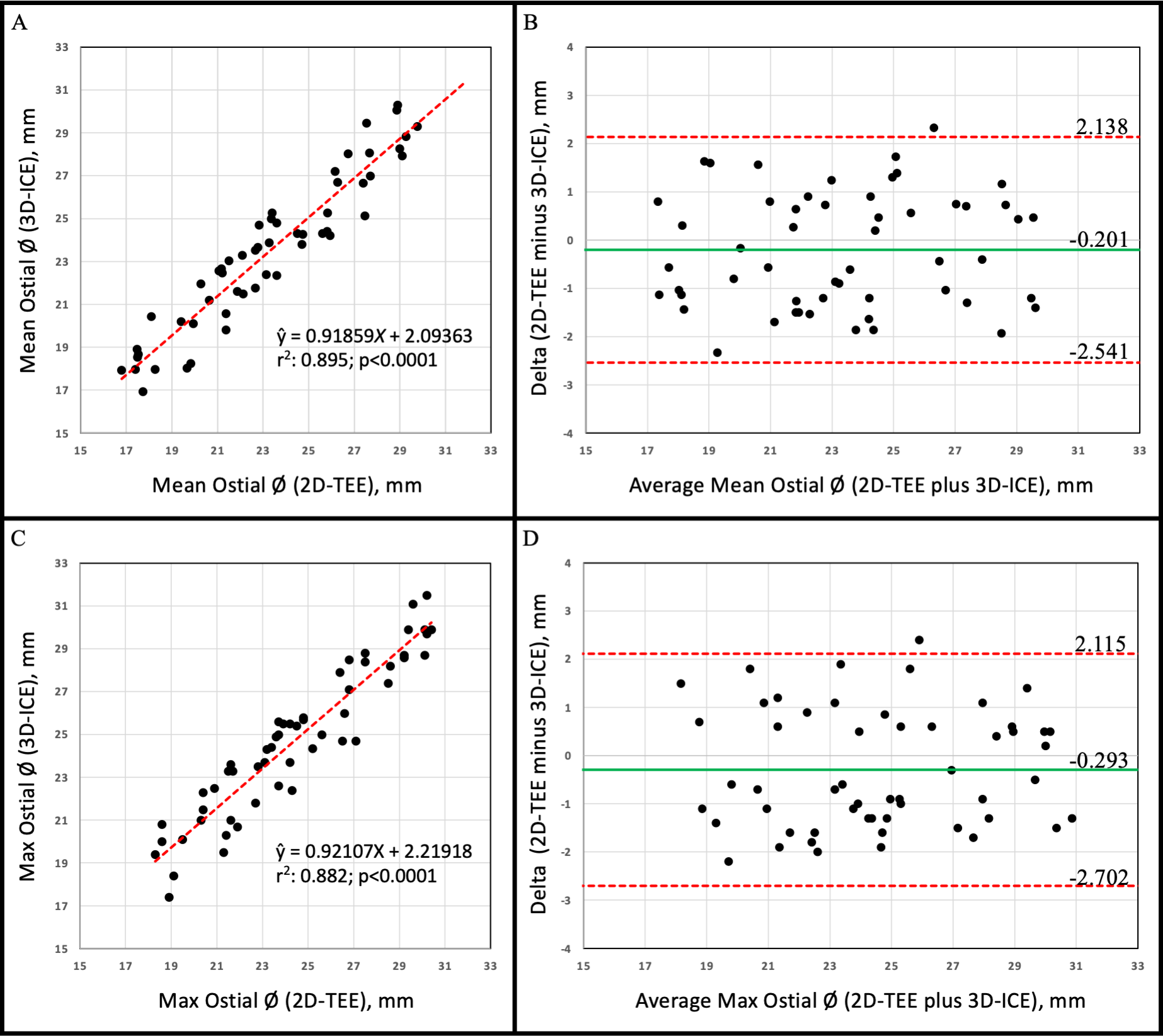

Supplement: euae010_Supplementary_Data [file euae010_supplementary_data.zip › Supplemental Fig.3.tiff]

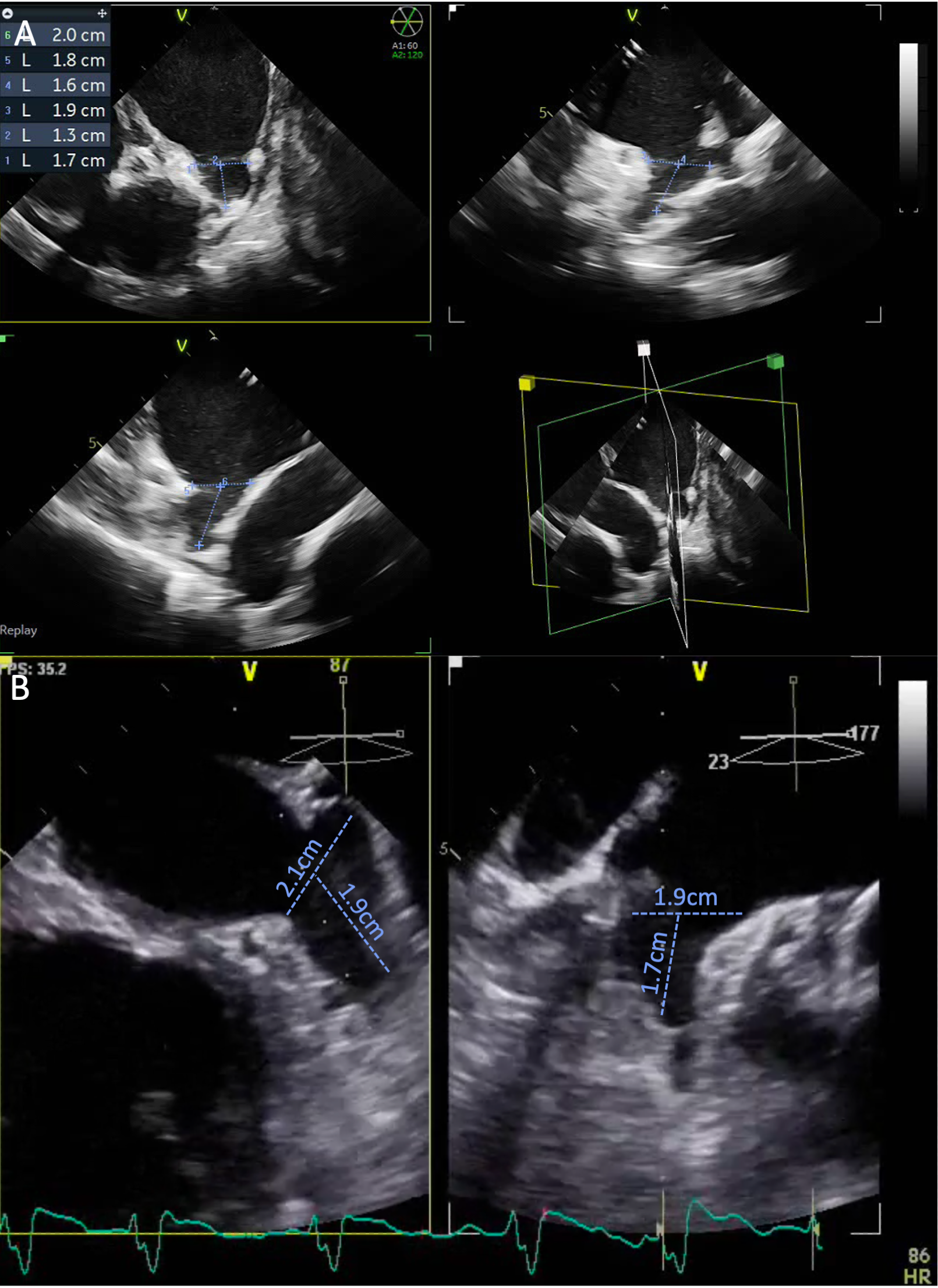

Supplement: euae010_Supplementary_Data [file euae010_supplementary_data.zip › Supplemental Fig.1.png]
